# Maternal death: Case definition and guidelines for data collection, analysis, and presentation of immunization safety data^☆^

**DOI:** 10.1016/j.vaccine.2016.03.042

**Published:** 2016-12-01

**Authors:** M. Patwardhan, L.O. Eckert, H. Spiegel, F. Pourmalek, C. Cutland, S. Kochhar, B. Gonik, The Brighton Collaboration Maternal Death Working Group

**Affiliations:** a Department of Obstetrics and Gynecology, Wayne State University School of Medicine, Detroit, MI, USA; b Department of Obstetrics and Gynecology, University of Washington, Seattle, WA, USA; c Henry Jackson Foundation, Bethesda, MD, USA; d School of Population and Public Health, University of British Columbia, Vancouver, British Columbia, Canada; e Medical Research Council: Respiratory and Meningeal Pathogens Research Unit, Johannesburg, Department of Science and Technology National Research Foundation, Vaccine Preventable Diseases, Faculty of Health Sciences, University of the Witwatersrand, Johannesburg, South Africa; f Global Healthcare Consulting, India

**Keywords:** Maternal death, Adverse event, Immunization, Vaccination

## 1 Preamble

### 1.1 Need for developing case definitions and guidelines for data collection, analysis, and presentation for maternal death as an adverse event following immunization

Every year, there are at least 200 million women who become pregnant worldwide. Of these, approximately 585,000 women were reported to die each year as a result of pregnancy and childbirth related complications before the end of the second millennium by the WHO. More recent data suggest approximately a fifty percent decline in maternal deaths worldwide [1]. Although these statistics are encouraging, the death of a mother in itself is catastrophic and has a strong detrimental influence on not only the newborn, but the entire family leading to a vast emotional, psychosocial and economic vacuum. The exact number of women who die each year secondary to pregnancy is still unknown, especially since the majority of these events happen in remote rural areas globally. There is a universal problem with underreporting and misclassification of maternal deaths. In the USA, maternal death rate from 1982 to 1996 was actually 1.3 to 3 times the rate based solely on vital statistics data [2].

It is important to establish pregnancy status, timing and cause of death to define maternal death, but there exists considerable variation in identifying symptoms, signs, diseases and methods of deciphering and reporting cause of death. This in turn negatively affects global coding of maternal deaths.

Considerable regional variations exist in the reporting of maternal deaths [3]. Developing a universal definition for this major event is crucial to prevent under reporting and to be able to assess global data with consistency and accuracy in light of the United Nations Millennium Development Goal (UN MDG) 5a that called for a 75% reduction in the maternal mortality ratio between 1990 and 2015. An expansion of the UN MDGs are Sustainable Development Goals which are a new set of universal targets to enable international economic, social and environmental development in the future. Within the Sustainable Development Goals, the obstetrics and gynecology community are urged to embrace opportunities and commit to playing a greater role in promoting the health of young couples and children in all countries and at all stages of economic development. Reduction in maternal mortality is one of the integrated indicators of monitoring progress in this area [4], [5]. Hence, aligning the definition of maternal mortality is needed to enhance accurate reporting and recording of progress.

#### 1.1.1 Vaccine considerations in pregnancy

Pregnancy is a state of generalized immune suppression geared to decrease an antigen-specific response against the semi-allogenic fetus [6]. This altered autoimmunity also contributes to increase in infectious disease severity e.g. pregnant women affected by influenza suffer worse health outcomes than non-pregnant [7], [8]. For maternal, fetal and neonatal protection, vaccination in pregnancy is encouraged and recommended. Live attenuated vaccines have not been utilized in pregnancy due to a theoretical possibility of infecting the fetus and the potential to cause an uncontrolled increment in the vaccine virus load leading to severe or fatal reactions. For this reason, inactivated vaccines are administered to pregnant women. But as with any drug or biological product, there is a possibility of an adverse reaction or a side effect to the administered vaccine in pregnancy such as a severe allergic reaction (anaphylaxis) which is rare but may be life-threatening.

An adverse reaction to a vaccine is defined as an untoward effect or any medical event caused by a vaccine that is extraneous to its primary purpose of producing immunity. It could be a true adverse reaction or just a temporally coincidental event, which may be indistinguishable from complications in pregnancy.

For the collection of adverse event following immunization (AEFI) information, the Brighton Collaboration Working Group recommends using AEFI report forms, following the existing Brighton Collaboration guidelines as well as the data elements as specified in the general drug safety guidelines by the International Conference on Harmonization of Technical Requirements for Registration of Pharmaceuticals for Human Use (http://www.ich.org), the ethical standards in research and reporting requirements for drug adverse events by the Council for International Organizations of Medical Sciences (CIOMS, http://www.cioms.ch). The Council for International Organizations of Medical Sciences (CIOMS) provides a case definition for a “Serious” AEFI as an AEFI that results in death, is life-threatening, requires in-patient hospitalization or prolongation of existing hospitalization, results in persistent or significant disability/incapacity, or is a congenital anomaly/birth defect (http://whqlibdoc.who.int/publications/2012/9789290360834_eng.pdf). Currently, there is no specific case definition for “Maternal death following immunization”.

An AEFI can be causally attributed to vaccine more readily if it occurs during a plausible time period following vaccination and if it corresponds to those previously associated with a particular vaccine [9], [10]. For more than one reasons, the temporal association of an AEFI may be more complicated in pregnancy than otherwise. For example, adverse events such as a mild inflammatory maternal response [11] and chorioamnionitis [12] have been demonstrated secondary to vaccination that have the potential to cause maternal morbidity and mortality. Also, a pregnant woman may suffer from obstetric complications such as hemorrhage, hypertensive disease of pregnancy, etc. that may mimic an AEFI. Thus a causative link to an adverse event may be difficult to establish. Also, careful interpretation of its temporal association during pregnancy is essential. Due to superior antenatal surveillance and optimal medical management available in some parts of the world now, a severe acute maternal morbidity (SAMM) or near-miss cases threatening a pregnant woman's survival but not leading to her death, may be averted in pregnancy for the time being. This may temporarily increase the rate of near-miss cases, but if complicated by death in the future, may be under reported as maternal deaths [13], [14], [15], [16], [17].

There are several studies that have tried to evaluate safety of immunization in pregnancy. Maternal death following immunization has not been reported in any of these. A review of reports to the Vaccine Adverse event reporting system (VAERS) conducted by the CDC did not report any maternal deaths from 1990 to 2009 after the administration of the influenza trivalent inactivated or the live attenuated vaccine. Similarly, no maternal deaths were identified after immunization with Hepatitis A and B vaccine [12], meningococcal polysaccharide protein conjugate vaccine [18], tetanus toxide, reduced diptheria toxoid and acellular pertussis vaccine [19].

According to the Tenth Revision of the International Classification of Diseases (ICD-10), a maternal death is the death of a woman temporal to pregnancy, childbirth or the postpartum period, irrespective of the cause of death (including accidental or incidental causes). Maternal deaths are further grouped based on the underlying cause of death such as direct and indirect causes and timing of death such as late maternal deaths. Progress toward improvement in maternal health status are measured by useful Maternal Mortality indicators such as Maternal mortality rate and ratio.

Direct Maternal Death- direct maternal death is death of a woman that results from obstetric complications of the pregnant state, which includes pregnancy, labor, and puerperium. Direct deaths can be caused by obstetric complications, unanticipated complications arising due to interventions, omissions, incorrect treatment, or from a chain of events resulting from a combination of these factors. WHO estimates that the greatest proportion of maternal deaths are due to direct causes which include hemorrhage (25%), sepsis (15%), abortion (13%), hypertensive disorders of pregnancy (12%), and obstructed labor (8%).

Indirect Maternal Death – indirect maternal death is death of a woman caused by non-obstetric conditions or diseases that may exist before pregnancy, but is aggravated by the physiologic effects of pregnancy. About 20% of maternal deaths are due to indirect causes. Some examples of preexisting illnesses that may be aggravated by pregnancy are heart disease, iron deficiency anemia, tuberculosis, hypertension, malaria, and diabetes mellitus.

Maternal death due to coincidental causes or other death – maternal death due to coincidental causes or other death is death during pregnancy, childbirth and the puerperium due to coincidental causes, e.g. suicide.

Maternal death due to unspecified causes – maternal death due to unspecified causes is death during pregnancy, childbirth and the puerperium where the underlying cause is unknown or was not determined.

Late maternal death – late maternal death is death of a woman from direct or indirect obstetric causes more than 42 days but less than one year after termination of pregnancy.

Maternal mortality rate maternal mortality rate is the number of maternal deaths per 1000 women of reproductive age (usually 15–49 years). This is an indicator of the risk of maternal death among women of reproductive age and provides an indication of the burden of maternal death in the adult female population.

Maternal mortality ratio maternal mortality ratio is the number of maternal deaths during a given time period per 100,000 live births during the same time period. This is a more commonly used indicator of risk of a woman of dying from a given pregnancy or her obstetric risk.

### 1.2 Methods for the development of the case definition and guidelines for data collection, analysis, and presentation for maternal death as an adverse events following immunization

Following the process described in the overview paper [21] as well as on the Brighton Collaboration Website http://www.brightoncollaboration.org/internet/en/index/process.html, the Brighton Collaboration Maternal death Working Group was formed in 2015 and included members of (clinical, academic, public health, industry) background. The composition of the working and reference group as well as results of the web-based survey completed by the reference group with subsequent discussions in the working group can be viewed at: http://www.brightoncollaboration.org/internet/en/index/working_groups.html.

To guide the decision-making for the case definition and guidelines, a literature search was performed using Medline, PubMed and the Cochrane Libraries, including the terms vaccines, vaccination, or immunization and adverse pregnancy outcomes and/or maternal death. The search resulted in the identification of 202 references. All abstracts were screened for possible reports of maternal death following immunization. Fifteen articles with potentially relevant material were reviewed in more detail, in order to identify studies using case definitions or, in their absence, providing clinical descriptions of the case material. Multiple general medical text books were also searched. Most publications were case reports of single cases. The terminology was very inconsistent and no specific definition for the adverse event was provided.

### 1.3 Rationale for selected decisions about the case definition of maternal death as an adverse event following immunization

According to the Tenth Revision of the International Classification of Diseases (ICD-10), a maternal death is the death of a woman while pregnant or within 42 days of termination of pregnancy, irrespective of the duration and site of the pregnancy, from any cause related to or aggravated by the pregnancy or its management but not from accidental or incidental causes. Since death following immunization would likely be classified as an “incidental cause”, it would not be reported as a “maternal death” and hence would be under reported. Further to this, a maternal death following immunization may not be recognized as such by the certifier and has a significant potential of being misclassified. To avoid these problems of under reporting or misreporting, it is essential to formulate a terminology that follows the universal definition of maternal mortality, is easily understood by the general public while reporting and also encompasses all the missed cases.

Using the broader ICD-10 terminology for recognizing maternal death as an adverse event following immunization, “Death of a woman during pregnancy, childbirth and the puerperium that is closely related temporally to an immunization event of the mother which is likely the single or contributory cause” may be a more appropriate and pertinent definition.

#### 1.3.1 *Timing post immunization*

The incidence of anaphylactic or severe allergic reactions to vaccines that have the potential to lead to death if unrecognized and untreated is reported to be extremely low with less than one case per million vaccine doses. More recent larger studies have not reported any deaths in the general population. Signs and symptoms from vasovagal reactions are common after vaccination, especially in pregnancy, which can often be mistaken for anaphylactic reactions [20]. Hence, correct diagnosis is important for accurate reporting. Further to correct diagnosis is precision in determining specific time frames for onset of symptoms of adverse allergic reactions following immunization [21], [22], [23].

The case definition is accompanied by guidelines which are structured according to the steps of conducting a clinical trial, i.e. data collection, analysis and presentation. Neither case definition nor guidelines are intended to guide or establish criteria for management of ill infants, children, or adults. Both were developed to improve data comparability.

### 1.4 Periodic review

Similar to all Brighton Collaboration case definitions and guidelines, review of the definition with its guidelines is planned on a regular basis (i.e. every three to five years) or more often if needed.

## 2 Case definition of maternal death

**Level 1**

Diagnosis of pregnancy established by any of the following documented criteria:

a. Ultrasound examination
b. Fetal heart tones
c. Positive serum or urine human chorionic gonadotropin pregnancy test
d. Delivery of a neonate or other products of conception (abortus, stillborn)

And

Death of the mother while pregnant or within 42 days of termination of pregnancy, irrespective of the duration and site of the pregnancy

And

Documentation of Cause of death as:

a. Direct: abortive outcome, hypertensive disorder, obstetric hemorrhage, pregnancy related infection, other obstetric complications, unanticipated complications
b. Indirect: non obstetric complications
c. Death during pregnancy, childbirth and the puerperium: other or coincidental

**Level 2**

Diagnosis of pregnancy established by any of the following criteria in the absence of Level 1 criteria:

a. LMP date
b. Serial Symphysio Fundal Height examinations

And

Death of the mother while pregnant or within 42 days of termination of pregnancy, irrespective of the duration and site of the pregnancy

And

Documentation of Cause of death as:

a. Direct: abortive outcome, hypertensive disorder, obstetric hemorrhage, pregnancy related infection, other obstetric complications, unanticipated complications
b. Indirect: non-obstetric complications
c. Death during pregnancy, childbirth and the puerperium: other or coincidental
d. Unspecified: unknown or undetermined

**Level 3**

Absence of Level 1 or 2 criteria for establishing diagnosis of pregnancy and:

a. Unsure LMP
b. No clinical examination documented

And

Death of the mother temporal to pregnancy, childbirth or the postpartum period when exact timing of death is unknown

And

Documentation of cause of death as:

a. Direct: abortive outcome, hypertensive disorder, obstetric hemorrhage, pregnancy related infection, other obstetric complications, unanticipated complications
b. Indirect: non obstetric complications
c. Death during pregnancy, childbirth and the puerperium: other or coincidental
d. Unspecified: unknown or undetermined.

## 3 Guidelines for data collection, analysis and presentation of maternal death

It was the consensus of the Brighton Collaboration Maternal death Working Group to recommend the following guidelines to enable meaningful and standardized collection, analysis, and presentation of information about maternal death. However, implementation of all guidelines might not be possible in all settings. The availability of information may vary depending upon resources, geographical region, and whether the source of information is a prospective clinical trial, a post-marketing surveillance or epidemiological study, or an individual report of maternal death. Also, as explained in more detail in the overview paper in this volume, these guidelines have been developed by this working group for guidance only, and are not to be considered a mandatory requirement for data collection, analysis, or presentation.

### 3.1 Data collection

These guidelines represent a desirable standard for the collection of data on availability following immunization to allow for comparability of data, and are recommended as an addition to data collected for the specific study question and setting. The guidelines are not intended to guide the primary reporting of maternal death to a surveillance system or study monitor. Investigators developing a data collection tool based on these data collection guidelines also need to refer to the criteria in the case definition, which are not repeated in these guidelines. The Brighton Collaboration has developed guidelines for data collection https://brightoncollaboration.org/public/resources/standards/guidelines.html; and data collection forms https://brightoncollaboration.org/public/resources/data-collection-forms.html.

Guidelines 1–40 below have been developed to address data elements for the collection of adverse event information as specified in general drug safety guidelines by the International Conference on Harmonization of Technical Requirements for Registration of Pharmaceuticals for Human Use (International Conference on Harmonisation of Technical Requirements for Registration of Pharmaceuticals for Human Use (ICH) (24), and the form for reporting of drug adverse events by the Council for International Organizations of Medical Sciences (Council for International Organizations of Medical Sciences (CIOMS) (25). These data elements include an identifiable reporter and patient, one or more prior immunisations, and a detailed description of the adverse event, in this case, of maternal death following immunization. The additional guidelines have been developed as guidance for the collection of additional information to allow for a more comprehensive understanding of maternal death following immunization.

#### 3.1.1 Source of information/reporter

For all cases and/or all study participants, as appropriate, the following information should be recorded:

(1) Date of report.
(2) Name and contact information of person reporting^2^ and/or diagnosing maternal death as specified by country-specific data protection law.
(3) Name and contact information of the investigator responsible for the subject, as applicable.
(4) Relation to the patient (e.g., immunizer [clinician, nurse], family member [indicate relationship], other).

#### 3.1.2 Vaccinee/control

##### 3.1.2.1 Demographics

For all cases and/or all study participants, as appropriate, the following information should be recorded:

(5) Case/study participant identifiers (e.g. first name initial followed by last name initial) or code (or in accordance with country-specific data protection laws).
(6) Date of birth, age, sex, ethnicity.

##### 3.1.2.2 Clinical and immunization history

For all cases and/or all study participants, as appropriate, the following information should be recorded:

(7) Past medical history, including hospitalizations, underlying diseases/disorders, pre-immunization signs and symptoms including identification of indicators for, or the absence of, a history of allergy to vaccines, vaccine components or medications; food allergy; allergic rhinitis; eczema; asthma.
(8) Any medication history (other than treatment for the event described) prior to, during, and after immunization including prescription and non-prescription medication as well as medication or treatment with long half-life or long term effect (e.g. immunoglobulins, blood transfusion and immunosuppressants).
(9) Immunization history (i.e. previous immunizations and any adverse event following immunization (AEFI)).

#### 3.1.3 Details of the immunization

For all cases and/or all study participants, as appropriate, the following information should be recorded:

(10) Date and time of immunization(s) and timing of immunization in relation with pregnancy: antepartum, intrapartum, or postpartum {antepartum: day or week of pregnancy or trimester, ascertainment method for time of conception (LMP, fundal height, ultrasound); postpartum: day or week postpartum}.
(11) Description of vaccine(s) (name of vaccine, manufacturer, lot number, dose (e.g. 0.25 mL, 0.5 mL, etc.) composition of any diluent administered separately or added to the vaccine, and number of dose if part of a series of immunisations against the same disease).
(12) The anatomical sites (including left or right side) of all immunisations (e.g. vaccine A in proximal left lateral thigh, vaccine B in left deltoid).
(13) Route and method of administration (e.g. intramuscular, intradermal, subcutaneous, and needle-free (including type and size), other injection devices).
(14) Needle length and gauge.

#### 3.1.4 The adverse event

(15) For all cases at any level of diagnostic certainty and for reported events with insufficient evidence, the criteria fulfilled to meet the case definition should be recorded.
  Specifically document:
(a) The issuer of death certificate (physician or other person authorized by the local law to issue death certificate) if a death certificate was issued,
(b) Place of death (hospital, health facility other than hospital, in transportation, home)
(c) If an autopsy was performed with results
(d) If a verbal autopsy was performed with results
(e) Cause of death as
i. Direct: abortive outcome, hypertensive disorder, obstetric hemorrhage, pregnancy related infection, other obstetric complications, unanticipated complications
ii. Indirect: non obstetric complications
iii. Death during pregnancy, childbirth and the puerperium: other or coincidental
iv. Unspecified: unknown or undetermined
(16) Clinical description of signs and symptoms preceding maternal death, and if there was medical confirmation of the event (i.e. patient seen by physician).
(17) Date/time of onset,^3^ diagnosis,^4^ clinical events^5^ and final outcome.^6^
(18) Concurrent signs, symptoms, and diseases.
(19) Measurement/testing
• Values and units of routinely measured parameters (e.g. temperature, blood pressure) – in particular those preceding maternal death;
• Method of measurement (e.g. type of thermometer, oral or other route, duration of measurement, etc.);
• Results of laboratory examinations, surgical and/or pathological findings and diagnoses if present.
(20) Objective clinical evidence supporting classification of the event.^7^
(21) Exposures other than the immunization 24 h before and after immunization (e.g. food, environmental) considered potentially relevant to the reported event.

#### 3.1.5 Miscellaneous/general

(22) The duration of surveillance for maternal death should be predefined based on
• Biologic characteristics of the vaccine e.g. live attenuated versus inactivated component vaccines;
• Biologic characteristics of the vaccine-targeted disease;
• Biologic characteristics of maternal death including patterns identified in previous trials (e.g. early-phase trials); and
• Biologic characteristics of the vaccinee (e.g. nutrition, underlying disease like immunosuppressive illness).
(23) The duration of follow-up reported during the surveillance period should be predefined likewise. It should aim to continue to resolution of the event.
(24) Methods of data collection should be consistent within and between study groups, if applicable.
(25) Follow-up of cases should attempt to verify and complete the information collected as outlined in data collection guidelines 1–24.
(26) Investigators of patients with maternal death should provide guidance to reporters to optimize the quality and completeness of information provided.
(27) Reports of maternal death should be collected throughout the study period regardless of the time elapsed between immunization and the adverse event. If this is not feasible due to the study design, the study periods during which safety data are being collected should be clearly defined.

### 3.2 Data analysis

The following guidelines represent a desirable standard for analysis of data on maternal death to allow for comparability of data, and are recommended as an addition to data analyzed for the specific study question and setting.

(28) Reported events should be classified in one of the following five categories including the three levels of diagnostic certainty. Events that meet the case definition should be classified according to the levels of diagnostic certainty as specified in the case definition. Events that do not meet the case definition should be classified in the additional categories for analysis.

**Event classification in 5 categories**^6^^6^

**Event meets case definition**

*(1)* Level 1: Criteria as specified in the Maternal death case definition
*(2)* Level 2: Criteria as specified in the Maternal death case definition
*(3)* Level 3: Criteria as specified in the Maternal death case definition
  **Event does not meet case definition**
  ***Additional categories for analysis***
*(4)* Reported maternal death with insufficient evidence to meet the case definition^8^
*(5)* Not a case of maternal death^9^

(29) The interval between immunization and reported maternal death could be defined as the date/time of immunization to the date/time of maternal death. If few cases are reported, the concrete time course could be analyzed for each; for a large number of cases, data can be analyzed in the following increments: see Table 1.
(30) The duration of events leading to maternal death could be analyzed as the interval between the date/time of onset^1^^1^ of the first symptoms and/or signs consistent with the definition and the final outcome.^5^^5^ Whatever start and ending are used, they should be used consistently within and across study groups.
(31) If more than one measurement of a particular criterion is taken and recorded, the value corresponding to the greatest magnitude of the adverse experience could be used as the basis for analysis. Analysis may also include other characteristics like qualitative patterns of criteria defining the event.
(32) The distribution of data (as numerator and denominator data) could be analyzed in predefined increments (e.g. measured values, times), where applicable. Increments specified above should be used. When only a small number of cases are presented, the respective values or time course can be presented individually.
(33) Data on maternal death obtained from subjects receiving a vaccine should be compared with those obtained from an appropriately selected and documented control group(s) to assess background rates of maternal death in non-exposed populations, and should be analyzed by study arm and dose where possible, e.g. in prospective clinical trials.

### 3.3 Data presentation

These guidelines represent a desirable standard for the presentation and publication of data on maternal death following immunization to allow for comparability of data, and are recommended as an addition to data presented for the specific study question and setting. Additionally, it is recommended to refer to existing general guidelines for the presentation and publication of randomized controlled trials, systematic reviews, and meta-analyses of observational studies in epidemiology (e.g. statements of Consolidated Standards of Reporting Trials (CONSORT), of Improving the quality of reports of meta-analyses of randomized controlled trials (QUORUM), and of Meta-analysis Of Observational Studies in Epidemiology (MOOSE), respectively) (26–28).

(34) All reported events of maternal death should be presented according to the categories listed in guideline 29.
(35) Data on possible maternal death events should be presented in accordance with data collection guidelines 1–27 and data analysis guidelines 28–33
(36) Data should be presented with numerator and denominator (n/N) (and not only in percentages), if available.
  Although immunization safety surveillance systems denominator data are usually not readily available, attempts should be made to identify approximate denominators. The source of the denominator data should be reported and calculations of estimates be described (e.g. manufacturer data like total doses distributed, reporting through Ministry of Health, coverage/population based data, etc.).
(37) The incidence of cases in the study population should be presented and clearly identified as such in the text.
(38) If the distribution of data is skewed, median and inter-quartile range (IQR) with mention of both the first and third quartiles (Q1 and Q3) are usually the more appropriate statistical descriptors than a mean. However, the mean and standard deviation should also be provided.
(39) Any publication of data on maternal death should include a detailed description of the methods used for data collection and analysis as possible. It is essential to specify:
• The study design;
• The method, frequency and duration of monitoring for maternal death;
• The trial profile, indicating participant flow during a study including drop-outs and withdrawals to indicate the size and nature of the respective groups under investigation;
• The type of surveillance (e.g. passive or active surveillance);
• The characteristics of the surveillance system (e.g. population served, mode of report solicitation);
• The search strategy in surveillance databases;
• Comparison group(s), if used for analysis;
• The instrument of data collection (e.g. standardized questionnaire, diary card, report form);
• Whether the day of immunization was considered “day one” or “day zero” in the analysis;
• Whether the date of onset^2^^2^ and/or the date of diagnosis^3^^3^ or date of registration/documentation/reporting was used for analysis
• Use of this case definition for maternal death, in the abstract or methods section of a publication^10^

## Figures and Tables

**Table 1:** Subjects with maternal death by interval to presentation.^a^

Interval	Day or week of pregnancy (trimester or postpartum)	Number	Percentage
0–72 h after immunization			
More than 72 h to 7 days after immunization			
More than 7 days to 30 days after immunization			
More than 30 days after immunization			
Time unit increments thereafter			

Total			

a Presentation should include death of the mother while pregnant or within 42 days of termination of pregnancy, irrespective of the duration and site of the pregnancy or death of the mother temporal to pregnancy, childbirth or the postpartum period when exact timing of death is unknown.

## Footnotes

2 If the reporting center is different from the vaccinating center, appropriate and timely communication of the adverse event should occur.

3 The date and/or time of onset is defined as the time post immunization, when the first sign or symptoms preceding maternal death occurred. This may only be possible to determine in retrospect.

4 The date of diagnosis of an episode is the day post immunization when the event met the case definition at any level.

5 E.g. Clinical events preceding maternal death, therapeutic interventions needed.

6 An AEFI is defined as serious by international standards if it meets one or more of the following criteria: (1) it results in death, (2) is life-threatening, (3) it requires inpatient hospitalization or results in prolongation of existing hospitalization, (4) results in persistent or significant disability/incapacity, (5) is a congenital anomaly/birth defect, (6) is a medically important event or reaction.

7 To determine the appropriate category, the user should first establish, whether a reported event meets the criteria for the lowest applicable level of diagnostic certainty, e.g. Level three. If the lowest applicable level of diagnostic certainty of the definition is met, and there is evidence that the criteria of the next higher level of diagnostic certainty are met, the event should be classified in the next category. This approach should be continued until the highest level of diagnostic certainty for a given event could be determined. Major criteria can be used to satisfy the requirement of minor criteria. If the lowest level of the case definition is not met, it should be ruled out that any of the higher levels of diagnostic certainty are met and the event should be classified in additional categories four or five.

8 If the evidence available for an event is insufficient because information is missing, such an event should be categorized as “Reported death with insufficient evidence to meet the case definition”.

9 An event does not meet the case definition if investigation reveals a negative finding of a necessary criterion (necessary condition) for diagnosis. Such an event should be rejected and classified as “Not a case of maternal death”.

10 Use of this document should preferably be referenced by referring to the respective link on the Brighton Collaboration website (http://www.brightoncollaboration.org).
