# Supplementary material for: Maternal death: Case definition and guidelines for data collection, analysis, and presentation of immunization safety data
Source: Vaccine. 2016 Dec 1;34(49):6077–83. doi: 10.1016/j.vaccine.2016.03.042 (PMC5139803; doi:10.1016/j.vaccine.2016.03.042)
Supplement: Supplementary file 1 [file mmc1.docx]

Rebuttal - Manuscript title:

Date:

|  | **Review comments** | | **Author reply** |
| --- | --- | --- | --- |
| **1.a. Do you agree with the rationale of the case definition?** | Yes | 13 |  |
|  | No | 0 |  |
|  | Do not know | 1 |  |
| 1.b. If no / do not know, explain why. | i might have missed it but where does 42 days come from? What is the rational? | | 6 weeks postpartum period |
| 1.c. Comments? | I would prefer if the WHO estimates are used. Also not sure why still the 500,000 deaths are treported when IHME and WHO report below 400,000. | | The 585,000 maternal deaths worldwide are WHO estimates. These were reported in 1996. Newer data suggests a 50% reduction which is a 2014 report and mentioned in the next sentence. |
| **2.a. Does the preamble clearly describe the intended use of the definition?** | Yes | 9 |  |
|  | No | 2 |  |
|  | Do not know | 0 |  |
| 2.b. If no / do not know, explain why. | You use outdated distribution of causes of maternal deaths. | | ?  The ICD-10 revision is the latest one. |
| 2.c. Comments? | see above, there are so many strudies indicating the underreporting, thus this could be much expanded and the EU lit be added.  I'm missing the definition of the MM ratio, per 100 000 live births or maybe I oberlooked | | ?  MM ratio can be included. |
| **2(2).a. Do you agree with the definition?** | Yes | 1 |  |
|  | No | 0 |  |
|  | Do not know | 2 |  |
| 2(2).b. If no / do not know, explain why. | The definition is so broad, that it is unclear whether any analysis with meaning can come from it. | | The latest definition of MM (ICD 10) is made in order to reduce mis or under reporting. An explanation is under section 1.3 |
|  | Taking in account that new medical methods to keep alive a women with a severe condition, and the fact that Interagency Group for Maternal Mortality Estimates suggested the inclusion of maternal death by sequel maternal death. Late Maternal Death and Sequel MD could be taking in account for the numerator of the Rate. At least for the period that covers the research | | ? |
| 2(2).c. Comments? | Level 1 appears to be the most sensitive diagnosis, while level 3 is the least sensitive -this is contrary to what you defined earlier. | | “-this is contrary to what you defined earlier.” - ? |
|  | You are suggesting the use of MM Rate instead MM Ratio the first one is not such commun and the second one. I think that you need to express the way to obtain the denominator (source) (Number ob women in reproductive age). | | We can include MM ratio as well. |
| **3.a. Do you agree with Level 1 of diagnostic certainty?** | Yes | 8 |  |
|  | No | 2 |  |
|  | Do not know | 1 |  |
| 3.b. If no / do not know, explain why. | I do not understand why incidental and accidental deaths are included under indirect??? | | ? |
|  | Indirect causes of death should include pre-existing maternal conditions such as heart diseases, renal disorders,etc. | | This is specified in the document. |
| 3.c. Comments? | The definition of direct maternal death as "abortive outcome... unanticipated complications" seems very odd and confusing. Why not use a standard definition rather than trying to list them? And are not severe malaria or a road traffic accident both 'unanticipated'? I don't undertsand why this has been used - i would replace it with a standard definition for all 3 levels (as well as amending the indirect definition to fit with WHO terminology). | | All the terms used are explained with definitions earlier in the document. |
| **4.a. Do you agree with Level 2 of diagnostic certainty?** | Yes | 10 |  |
|  | No | 1 |  |
|  | Do not know | 0 |  |
| 4.b. If no / do not know, explain why. | again, why including incidental | | ? |
| 4.c. Comments? |  | |  |
| **5.a. Do you agree with Level 3 of diagnostic certainty?** | Yes | 10 |  |
|  | No | 1 |  |
|  | Do not know | 0 |  |
| 5.b. If no / do not know, explain why. | somehow easier to use WHO terminology "regardless the cause" | | The newer ICD -10 coding suggests the use of the different terms. |
| 5.c. Comments? |  | |  |
| **6.a. Do you agree with the footnotes of the case definition?** | Yes | 7 |  |
|  | No | 1 |  |
|  | Do not know | 2 |  |
| 6.b. If no / do not know, explain why. | ? | |  |
|  | My observation is that this standard case definition combines two case definitions whose definitions are to some extent already agreed upon I e maternal mortality and AEFI.  My take would be that we marry the two paying attention to the details each definition for example 6 weeks features very well in the definition of maternal mortality and I think 4 weeks as timing post vaccination need to feature in the definition Suggestion  In the definition I wish to suggest as follows: we replace the word Immunization with vaccination since immunization is the intended outcome and there may be errors with vaccination process.  Maternal SCD  Death of a woman during pregnancy, child birth and puerperium that is closely related temporally to the immunization event of the mother which is the likely single or contributory cause  1 How close is this temporal closeness? In terms of time, if so how long, 4 weeks? 6 weeks? etc, this temporal closeness needs to be a defined a little more for preciseness 2 which is the likely single or contributory cause this presupposes a cause/effect relationship between the vaccine and the maternal death/AEFI which is a conclusion arrived at after investigation of the AEFI but not at detection level, this needs to be clarified.  How do you determine the likelihood of being the single or contributory cause? Doesn’t this signify biase at the level of detection rather than investigation of the AEFI? The likely result will be under reporting of the AEFI, and lack of standardization hence the implementability of the SCD and comparability of data across reporting centers  In addition this has far-reaching implications to the EPI program more so at such a time tolerance to vaccination is rising in some settings. Litigation with costs will follow. | | Immunization – was used in the Brighton template  Temporal closeness is not defined in days or weeks to allow maximum reporting of the event and reduce mis or under reporting.  Point 29 gives time increments after immunization for maternal death  The likelihood of immunization being a cause for maternal death may be determined immediately in some cases i.e severe anaphylaxis after immunization while in some cases may be determined after investigation or may not be determined at all. The key would be that a maternal death is determined to be in association with an immunization event in a temporal time frame. If it can be determined with some level of certainty that the maternal death was in fact secondary to immunization, then it would fall under the category of maternal death after immunization. |
| 6.c. Comments? |  | |  |
| **7.a. Is the definition applicable in your setting?** | Yes | 7 |  |
|  | No | 1 |  |
|  | Do not know | 2 |  |
| 7.b. If no / do not know, explain why. | This may have far-reaching implications to the EPI program more so at such a time tolerance to vaccination is rising in some settings. Litigation with costs will follow. | |  |
|  | Only Tetanus toxoid is given during pregnancy. Cerification of maternal death is not proper mostly. Many mothers still deliver without skilled birth attendants. | | ? |
| 7.c. Comments? | Any one of the 3 levels of case definitions will be applicable depending on the rural/urban setting and availability/utilization of health services related to MCH | | ? |
| **8.a. Do you agree with the guidelines?** | Yes | 9 |  |
|  | No | 1 |  |
|  | Do not know | 3 |  |
| 8.b. If no / do not know, explain why. | why not using the WHO documentation on how to report maternal deaths? | |  |
|  | Time in relation to the immunization program needs to be collected as well, is it during morning or evening sessions, as timing in relation to the pregnancy or termination will have been collected from time of onset of the AEFIs, duration after the vaccination. Data also needs to be collected on cold chain system management, adherence to infection prevention processes, reconstitution processes Suggested SCD for Maternal death as an AEFI Death of a woman during pregnancy, child birth and puerperium 4 weeks prior to the vaccination event of the mother but may not have a cause effect relationship with the vaccine. | | “Death of a woman during pregnancy, child birth and puerperium 4 weeks prior to the vaccination event of the mother but may not have a cause effect relationship with the vaccine.” – The time interval is described as temporal so as to include majority cases that may be missed. The “which is the likely single or contributory cause” – is used so that the inclusion of cases are more specific to maternal death after immunization. The ICD-10 defines “underlying cause of death” as a condition that initiated morbid chain of events leading to death with a single identified cause as specific as possible. |
| 8.c. Comments? | The time interval from immunisation to death (Pg17) : More than 30 days after immunisation, may have no association between the two events, | | “More than 30 days after immunisation,” – this would include those rare cases, which after immunization resulted in prolonged morbidity/organ failure resulting in late deaths. |
|  | Please add ethnicity to vaccine/control demographics. Very important variable. Also need to add to non-reassuring fetal status guidelines. | | Added |
| **9. Any general comments?** | The guidelines seem to be inadequate on infection control practices that may also predispose the clients to AEFIs | |  |
|  | There is word of caution that an attempt should not be made to define or mainstream the word ‘maternal death following immunization’. This term would be a great misnomer providing a false impression that maternal deaths are common phenomenon after vaccination/immunization, which they are not. Please delete all such mentions.  Page 5, instead of the use of maternal mortality rate, please adhere to the standard maternal mortality ratio (MMR). | | Use of Maternal death following immunization – What do we all think about its inclusion and the possibility of litigation?   MM ratio is added. |
|  | In our setting immunization in pregnancy means Tetanus toxoid. | |  |
|  | Better initiative as far as AEFI of immunization is concerned.Need based,relevant and timely step indeed. | | ? |
| **7. Any other comments you would like to share?** |  | |  |
| **3.a. Based on your study setting and the procedures / diagnostic requirements for the different levels of certainty in the case definition, would you be able to obtain all levels of certainty?** | Yes | 1 |  |
|  | No | 1 |  |
|  | Do not know | 1 |  |
| 3.b. If no / do not know, explain which level(s) would not be applicable and why (e.g. not having all procedures available in countries where study is done / retrospective assessment of charts would not allow information / exclusion / negative criteria to be found consistently) | why would we want to attain all levels of certainty? the most specific and sensitive are the gold standard. LMIC probably cannot provide the most sensitive level definitions. | | We should aim at obtaining Level 1; if not, then Level 2; if not then Level 3 |
|  | There are a lot of items (ex.3.1.3) that probably we can´t obtain. | |  |
| 3.c. Comments? |  | |  |
